# Modulating limbic circuits in temporal lobe epilepsy: impacts on seizures, memory, mood and sleep

**DOI:** 10.1093/braincomms/fcaf106

**Published:** 2025-04-07

**Authors:** Vaclav Kremen, Vladimir Sladky, Filip Mivalt, Nicholas M Gregg, Benjamin H Brinkmann, Irena Balzekas, Victoria Marks, Michal Kucewicz, Brian Nils Lundstrom, Jie Cui, Erik K St Louis, Paul Croarkin, Eva C Alden, Boney Joseph, Julie Fields, Karla Crockett, Jindrich Adolf, Jordan Bilderbeek, Dora Hermes, Steven Messina, Kai Joshua Miller, Jamie Van Gompel, Timothy Denison, Gregory A Worrell

**Affiliations:** Department of Neurology, Bioelectronics Neurophysiology and Engineering Laboratory, Mayo Clinic, Rochester, MN 55905, USA; Czech Institute of Informatics, Robotics, and Cybernetics, Czech Technical University in Prague, Prague 16000, Czech Republic; Department of Neurology, Bioelectronics Neurophysiology and Engineering Laboratory, Mayo Clinic, Rochester, MN 55905, USA; Faculty of Biomedical Engineering, Czech Technical University in Prague, Kladno 27201, Czech Republic; Department of Neurology, Bioelectronics Neurophysiology and Engineering Laboratory, Mayo Clinic, Rochester, MN 55905, USA; Department of Biomedical Engineering, Faculty of Electrical Engineering and Communication, Brno University of Technology, Brno 61600, Czech Republic; Department of Neurology, Bioelectronics Neurophysiology and Engineering Laboratory, Mayo Clinic, Rochester, MN 55905, USA; Department of Neurology, Bioelectronics Neurophysiology and Engineering Laboratory, Mayo Clinic, Rochester, MN 55905, USA; Department of Physiology and Biomedical Engineering, Mayo Clinic, Rochester, MN 55905, USA; Department of Neurology, Bioelectronics Neurophysiology and Engineering Laboratory, Mayo Clinic, Rochester, MN 55905, USA; Department of Physiology and Biomedical Engineering, Mayo Clinic, Rochester, MN 55905, USA; Department of Neurology, Bioelectronics Neurophysiology and Engineering Laboratory, Mayo Clinic, Rochester, MN 55905, USA; Department of Physiology and Biomedical Engineering, Mayo Clinic, Rochester, MN 55905, USA; Department of Neurology, Bioelectronics Neurophysiology and Engineering Laboratory, Mayo Clinic, Rochester, MN 55905, USA; BioTechMed Center, Brain and Mind Electrophysiology Lab, Multimedia Systems Department, Faculty of Electronics, Telecommunication and Informatics, Gdansk University of Technology, Gdansk 80-233, Poland; Department of Neurology, Bioelectronics Neurophysiology and Engineering Laboratory, Mayo Clinic, Rochester, MN 55905, USA; Department of Neurology, Bioelectronics Neurophysiology and Engineering Laboratory, Mayo Clinic, Rochester, MN 55905, USA; Divisions of Sleep Neurology and Pulmonary and Critical Care Medicine, Departments of Neurology and Medicine, Center for Sleep Medicine, Mayo Clinic, Rochester, MN 55905, USA; Departments of Psychiatry and Psychology, Mayo Clinic, Rochester, MN 55905, USA; Departments of Psychiatry and Psychology, Mayo Clinic, Rochester, MN 55905, USA; Department of Neurology, Bioelectronics Neurophysiology and Engineering Laboratory, Mayo Clinic, Rochester, MN 55905, USA; Departments of Psychiatry and Psychology, Mayo Clinic, Rochester, MN 55905, USA; Department of Neurology, Bioelectronics Neurophysiology and Engineering Laboratory, Mayo Clinic, Rochester, MN 55905, USA; Czech Institute of Informatics, Robotics, and Cybernetics, Czech Technical University in Prague, Prague 16000, Czech Republic; Department of Physiology and Biomedical Engineering, Mayo Clinic, Rochester, MN 55905, USA; Department of Physiology and Biomedical Engineering, Mayo Clinic, Rochester, MN 55905, USA; Department of Radiology, Mayo Clinic, Rochester, MN 55905, USA; Department of Neurologic Surgery, Mayo Clinic, Rochester, MN 55905, USA; Department of Neurologic Surgery, Mayo Clinic, Rochester, MN 55905, USA; Department of Engineering Science, Medical Research Council Brain Network Dynamics Unit, University of Oxford, Oxford OX3 7DQ, UK; Department of Neurology, Bioelectronics Neurophysiology and Engineering Laboratory, Mayo Clinic, Rochester, MN 55905, USA; Department of Physiology and Biomedical Engineering, Mayo Clinic, Rochester, MN 55905, USA

**Keywords:** electrical brain stimulation, epilepsy comorbidities, intracranial EEG, artificial intelligence and machine learning

## Abstract

Temporal lobe epilepsy is a common neurological disease characterized by recurrent seizures that often originate within limbic networks involving amygdala and hippocampus. The limbic network is involved in crucial physiologic functions involving memory, emotion and sleep. Temporal lobe epilepsy is frequently drug-resistant, and people often experience comorbidities related to memory, mood and sleep. Deep brain stimulation targeting the anterior nucleus of the thalamus (ANT-DBS) is an established therapy for temporal lobe epilepsy. However, the optimal stimulation parameters and their impact on memory, mood and sleep comorbidities remain unclear. We used an investigational brain sensing-stimulation implanted device to accurately track seizures, interictal epileptiform spikes (IES), and memory, mood and sleep comorbidities in five ambulatory subjects. Wireless streaming of limbic network local field potentials (LFPs) and subject behaviour were captured on a mobile device integrated with a cloud environment. Automated algorithms applied to the continuous LFPs were used to accurately cataloged seizures, IES and sleep-wake brain state. Memory and mood assessments were remotely administered to densely sample cognitive and behavioural response during ANT-DBS in ambulatory subjects living in their natural home environment. We evaluated the effect of continuous low-frequency and duty cycle high-frequency ANT-DBS on epileptiform activity and memory, mood and sleep comorbidities. Both low-frequency and high-frequency ANT-DBS paradigms reduced seizures. However, continuous low-frequency ANT-DBS showed greater reductions in IES, electrographic seizures and better sleep and memory outcomes. These results highlight the potential of synchronized brain sensing and dense behavioural tracking during ANT-DBS for optimizing neuromodulation therapy. While studies with larger patient numbers are needed to validate the benefits of low-frequency ANT-DBS, these findings are potentially translatable to individuals currently implanted with ANT-DBS systems.

## Teaser

Kremen *et al*. report that both continuous low-frequency and duty cycle high-frequency anterior nucleus of the thalamus deep brain stimulation (ANT-DBS) reduced seizures in five ambulatory subjects with temporal lobe epilepsy. However, low-frequency ANT-DBS showed greater reductions in interictal epileptiform spikes, electrographic seizures and better sleep and memory outcomes.

## Graphical Abstract

**Graphical Abstract:**
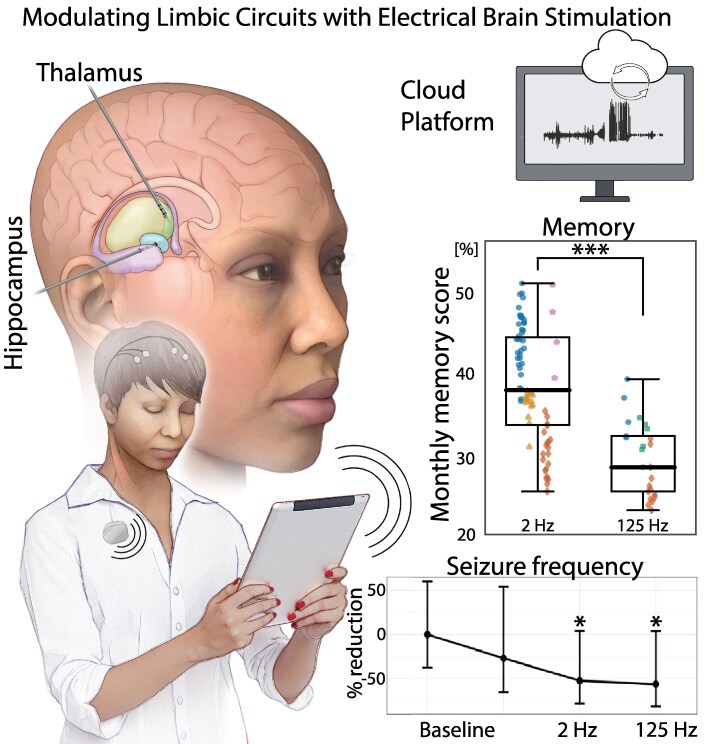

## Introduction

Epilepsy is a prevalent neurological condition marked by repeated seizures, affecting over 50 million individuals globally^1,2^ Besides the seizures themselves, the lives of people with epilepsy (PWE) are often disrupted by associated psychiatric and neurological issues.^3^ Epilepsy is a network circuit disorder with dysregulation of specific brain networks underlying the generation of sporadic seizures and chronic comorbidities.^3-5^ While anti-seizure medications represent the cornerstone of treatment for epilepsy, more than one-third of PWE are resistant to these drugs^6^ and seizures persist in experiencing despite medications.

Mesial temporal lobe epilepsy (mTLE) is among the most prevalent types of epilepsy, characterized by focal seizures that arise from limbic circuits^7,8^ specifically impacting amygdala, hippocampus (HPC) and parahippocampal neocortex functions. It is frequently drug-resistant and mood, memory and sleep (MMS) disturbances^9,10^ are common given the limbic circuit origin. Resective or ablative surgical procedures targeting mesial temporal structures are proven treatments for drug-resistant mTLE.^10,11^ For many people with mTLE, however, surgical intervention is not a viable option since removing the neural pathways responsible for their seizures may negatively impact normal functions.^10,12-14^ The limbic circuitry involved in mTLE also plays a crucial role in normal cognitive function, memory and emotional regulation, apart from causing intermittent, disabling seizures. People with normal structural imaging,^12^ bilateral mTLE,^15^ or high baseline memory performance^13^ pose a particular challenge for safe and effective destructive epilepsy surgery, inspiring interest in non-destructive, reversible therapeutic approaches such as electrical brain stimulation.^16^

Deep brain electrical stimulation targeting the anterior nucleus of the thalamus (ANT-DBS) is a proven neuromodulation therapy for drug-resistant focal epilepsy.^17,18^ The pivotal SANTE trial was a landmark multi-center, placebo-controlled, double-blind clinical trial that showed duty cycle, high-frequency (145 Hz; 1 min on and 5 min off) ANT-DBS reduced patient-reported seizures. The majority of individuals in the SANTE trial had TLE (66%), and the use of high-frequency (HF-ANT) stimulation was motivated by animal research showing it increased seizure threshold^19-21^ and disrupted seizure propagation.^16,22^

High-frequency ANT-DBS resulted in a 40.4% median reduction in patient-reported seizures from baseline, compared with a 14.5% reduction in the control non-stimulation group (*P* < 0.05) over a three-month period. In the subsequent 5-year open-label phase, the median seizure reduction reached 69%, and 16% of participants had at least one 6-month seizure-free period.^18^ The ANT^23,24^ is an important limbic network node, and ANT-DBS can impact MMS. In the SANTE trial, participants receiving HF-ANT reported depressive mood (14.8% versus 1.8%) and memory impairment (13.0% versus 1.8%) symptoms more frequently than the non-stimulated control group.^17^ Interestingly, at the group level, mood and memory changes were not captured by standard neuropsychological (NP) assessments.^25^ An independent study investigating SANTE ANT-DBS did not find a group level mood change during stimulation treatment, but reported individual patients with histories of depression may experience sudden depressive symptoms with ANT-DBS that can be alleviated by adjusting stimulation parameters.^26^ In addition, a study investigating cognitive changes with ANT-DBS showed fewer symptoms of executive dysfunction with ANT-DBS, and those with significant seizure reduction showed improved verbal learning.^27^

The effect of ANT-DBS on sleep has received less attention, but it is known SANTE ANT-DBS can disrupt sleep.^28,29^

These results highlight the potential importance of quantitatively tracking seizures and MMS comorbidities during ANT-DBS. Relatively little is known about ANT-DBS parameter optimization.^30,31^ Unfortunately, patient-reported seizure diaries are known to be inaccurate,^32-35^ and LFP based seizure catalogs are limited on currently available clinical devices.^36-38^ We propose that without accurate seizure catalogs and appropriately dense behavioural tracking of MMS comorbidities, optimizing neuromodulation therapy will remain challenging.

Inaccurate seizure diaries and inadequate sampling of common MMS comorbidities remain fundamental gaps in clinical epileptology. Informed by our previous studies in humans and pet canines with epilepsy^35,39-41^ using streaming hippocampal local field potentials (LFPs) for accurate seizure and interictal epileptiform spike (IES) detection, and motivated to collect continuous ecologically realistic behavioural data in ambulatory subjects, we developed a neurotechnology platform to accurately catalog seizures, IES and MMS comorbidities. The BrainRISE (Brain Restoration and Intelligent Stimulation Ecosystem) platform enables continuous bi-directional communication between an implanted sensing-stimulation device, local mobile devices and cloud computing environment using wireless cellular and internet protocols. Remote assessments of MMS performed on the subjects’ mobile devices are synchronized with limbic network LFPs. Automated algorithms are used to create accurate catalogs of seizures, IES and sleep-wake brain states.

In the SANTE trial, HF-DBS was proven to reduce seizures, but it can exacerbate MMS comorbidities. Low-frequency (LF-DBS) has also been shown to suppress seizure thresholds in rodent^42-45^ models and in humans.^46,47^ A functional MRI study comparing acute HF-DBS and LF-DBS has shown differential modulation of cortical and subcortical networks, with HF-DBS producing widespread cortical and subcortical deactivation sparing limbic and default mode networks and LF-DBS deactivating default mode network and limbic networks.^48^ To directly compare the impact of LF-DBS and HF-DBS on IES, seizures and MMS comorbidities in mTLE, we used the BrainRISE platform and a novel investigational sensing-stimulation implantable device with four electrode leads targeting bilateral amygdala-hippocampus (HPC-AMG) and ANT.

## Materials and methods

### Study and participant details

**Figure 1:**
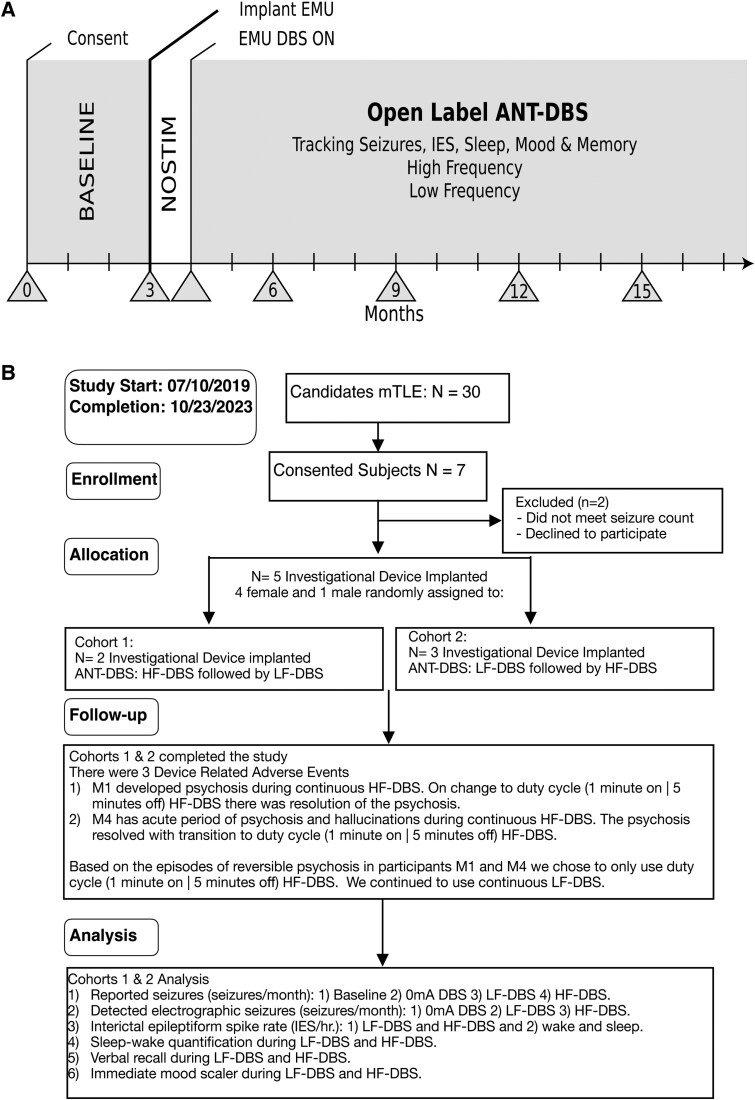
**Safety and feasibility trial of papez circuit tracking and control**. (**A**) The grey triangles denote in person office and hospital visits. Time 0 is the beginning of the study at time of consent for the study. Time 0 to 3 months is the diary phase where participants demonstrate use of EPA application to catalog seizures and medications (BASELINE). Data on charging and maintaining devices was collected throughout the study. At 3 months participant implanted with the INSS system with no DBS (NOSTIM). In the weeks following implant (∼4 weeks post-implant) participants were admitted to EMU for recording simultaneous PSG and intracranial EEG, i.e. LFPs, from HPC and ANT. The participant leaves the EMU with low-frequency DBS targeting the ANT-DBS. After 3–6 months of optimization of low-frequency ANT-DBS the participant transitioned to high-frequency duty cycle ANT-DBS. NP data, quality of life, mood and seizure severity were collected at specified in person clinic follow-ups. (**B)** Consolidated Standards of Reporting Trials (CONSORT). HF-DBS, high-frequency ANT-DBS; IES, interictal epileptiform spikes; LF-DBS, low-frequency ANT-DBS; mTLE, mesial temporal lobe epilepsy; M1–M5 identification labels of participants in the study.

Between the study start (10 July 2019) and the study completion (23 October 2023) seven people with drug-resistant bilateral mTLE were consented, and five people (Table 1 and Fig. 1) were implanted with an investigational neural sensing and stimulation device under FDA IDE: G180224 and Mayo Clinic IRB: 18-005483 Human Safety and Feasibility Study of Neurophysiologically Based Brain State Tracking and Modulation in Focal Epilepsy. The participants provided written consent in accordance with IRB and FDA requirements. Study registration https://clinicaltrials.gov/ct2/show/NCT03946618. The FDA IDE safety feasibility trial was approved for 10 patients. However, due to slow enrollment during the COVID-19 pandemic only five subjects were implanted with the investigational device.

**Table 1:** Patient demographics and clinical information

Participant	Age, hand (L/R), gender (M/F), onset age and DRE (years.)	Risk factor (RF) medical Hx: surgical Hx:	TL seizure types	ASM 1) Current 2) Failed ASM	EEG 1) Scalp 2) Intracranial	MRI PET SPECT
M1	55–60 y.o.; RF. Onset 5–10. y.o. DRE: 36 years. EMU: scalp EEG and sEEG.	RF: TBI Medical: Anxiety and depression Surgical: None	FAS, FIAS, Rare FBTC	1) GBP, CZP 2) LEV, OXC PHT, CBZ	1) B.IES & Szs. 2) B.HPC IES and Szs	MRI: Normal PET: N/A SPECT: N/A
M2	20–25 y.o.; RF. Onset 5–10 y.o. DRE 3 years. EMU: scalp EEG only	RF: None Medical: Anxiety, Depression, DM. Surgical: L.ATL	FAS, FIAS, Rare FBTC	1) LCM, CZP, 2) LEV, PHT, CBZ, OXC	1) B. IES and Sz. 2) N/A	MRI: L.ATL PET: N/A SPECT: N/A
M3	40–45 y.o.; RF. Onset 30–35 y.o. DRE 10 years. EMU: scalp EEG & iEEG	RF: None Medical: Anxiety, Depression, GAD-65 positive serum/CSF Surgical: VNS	FAS, FIAS, Rare FBTC	1) CZP, LEV, CNB 2) LZP, CBZ, PHT, TPX, LCM, CBD	1) B.IES and Szs. 2) B.HPC IES and Szs	MRI: Normal PET: N/A SPECT: N/A
M4	35–40 y.o.; RF. Onset 0–5 y.o. DRE 7 years. EMU: scalp EEG and sEEG	RF: None Medical: Anxiety, depression, GAD-65 positive serum/CSF Surgical: None	FAS, FIAS, Rare FBTC	1) OXC, LEV 2) LCM, CBZ, PHT, LGT	1) B.IES and Szs. 2) B. HPC IES and Szs	MRI: Normal PET: N/A SPECT: N/A
M5	30–35 y.o. RM. Onset 20–25 y.o. DRE 5 years. EMU: scalp EEG only	RF: TBI and Family history of epilepsy Medical: Anxiety, depression Surgical: None	FAS, FIAS, Rare FBTC	1) LCM, VPA 2) LEV, LGT, PHT, CBZ	1) B.IES and Szs. 2) N/A	MRI: L. HPC atrophy and incr. T2 signal PET: N/A SPECT: N/A

The patients had mTLE and HPC seizures. All patients had bilateral independent IES and seizures in the Phase-1 evaluation and M1, M3 and M4 had invasive stereo-EEG (sEEG) evaluations and had bilateral independent IES and seizures.

ATL, anterior temporal lobectomy; B, bilateral; CBZ, carbamazepine; CNB, cenobamate; CZP, clonazepam; DBS, deep brain electrical stimulation; DRE, drug-resistant epilepsy; EEG, electroencephalography; FAS, focal aware seizures; FBTC, focal to bilateral tonic-clonic; FIAS, focal impaired awareness seizures; GBP, gabapentin; HPC, hippocampus; IES, interictal epileptiform spikes; Incr, increased; LCM, lacosamide; LEV, levetiracetam; LGT, lamotrigine; LZP, lorazapam; M/F, male/female; OXC, oxcarbazepine; PHT, phenytoin; R/L, right/left; Sz, seizures; TBI, traumatic brain injury; TL, temporal lobe; VNS, vagus nerve stimulation; VPA, valproic acid; y.o., years old.

#### The study protocol

Participant inclusion required demonstration of drug-resistant mTLE. For inclusion, the patients must have had bilateral independent left and right temporal lobe onset seizures, or seizures from the dominant temporal lobe. Participants were required to have three or more disabling seizures per month as demonstrated on a mobile epilepsy patient assistant application (EPA) diary (supplementary data).

Participants were required to have disabling focal aware seizures (FAS), focal impaired awareness seizures (FIAS), or focal to bilateral tonic-clonic seizures (FBTC). Seven participants were consented, and five successfully completed the seizure diary baseline and were implanted with the investigational implantable neural sensing and stimulation device (INSS). Participants remained on stable medication regimes over the course of the study, except for participants M3 and M5, where it was determined clinically necessary to modify anti-seizure medications. Participant M3 entered the trial on a high-dose oral diazepam regimen that was ultimately discontinued after device implant due to worsening sedation and fatigue. Participant M5 was discovered to have frequent electrographic seizures after implant of the device, and his lacosamide and valproic acid were increased prior to initialing ANT-DBS.

We compared continuous low-frequency and duty cycle high-frequency ANT-DBS and the effect on long-term catalogs of IES, seizures and quantitative MMS measures.

#### Evaluations prior to enrollment

All participants had: (i) an epilepsy monitoring unit (EMU) evaluation to record habitual seizures with scalp video-EEG (31 to 76 scalp electrodes. (ii) 7T structural and functional MRI (unless 7T MRI was contraindicated, then 3T or 1.5T). The fMRI is to determine language lateralization. (iii) NP evaluation. (iv) Presentation at a multidisciplinary epilepsy surgery conference (v) Based on the results of the pre-surgical evaluation and multidisciplinary epilepsy surgery conference patients may proceed to Wada study to lateralize language and access risk of memory impairment with surgery. Furthermore, patients may undergo invasive Phase II monitoring with intracranial electrodes and prolonged video intracranial EEG monitoring for precise localization of their seizures if necessary. Patients with bilateral mesial temporal lobe seizures or dominant mesial temporal lobe seizures were candidates for this study.

#### Evaluations repeated during study

Comprehensive outcome assessments were obtained during scheduled outpatient clinic visits at baseline prior to implant, 3–6 months post-implant and 9–15 months post-implant. The instruments collected include the following: (i) Mood and Suicidality Assessments collected during office visits. (ii) Quality of Life Assessments during office visits. (iii) Seizure Severity Assessment during office visits. (iv) NP assessments during office visits.

#### BrainRISE (brain restoration, intelligent sensing-stimulation ecosystem) platform

**Figure 2:**
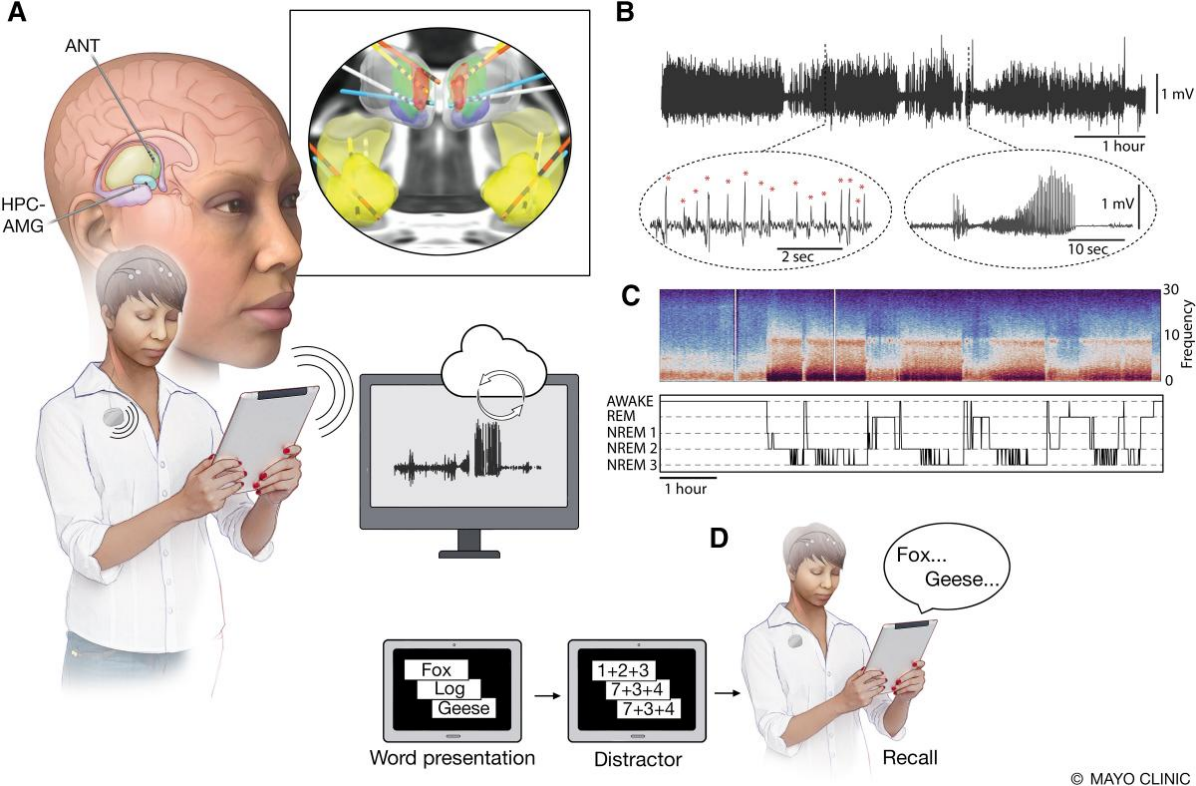
**Monitoring brain electrophysiology and behaviour in ambulatory humans living in their home environment**. (**A**) The INSS device enables wireless streaming of brain LFP to a mobile device running the EPA. The EPA application orchestrates integration and synchronization of multiple wireless devices and provides a system for bidirectional communication between devices, participants, and the remote clinical team. In five participants with mTLE, limbic circuit network nodes were implanted with four, 4-contact leads targeting bilateral HPC-AMG and ANT (inset view). The sensing-stimulation lead extensions were tunneled under the skin to the INSS device implanted in a surgically created pocket over the left pectoralis muscle. The INSS device supports bidirectional wireless communications and streaming of 4-bipolar LFP channels to a mobile device via a relay telemetry device typically worn by the individual. Bidirectional wireless communication between the INSS, mobile device and cloud environment enables analysis of synchronized brain electrophysiology, patient inputs and behaviour. Automatically or manually triggered behavioural surveys are presented on the mobile device. (**B)** Representative LFP recorded from epileptogenic HPC. Automated narrow Artificial Intelligence algorithms were deployed to capture pathological IES and electrographic seizure discharges (Sz). Circled insets highlight automated IES and Sz detections in an ambulatory participant (M1) in their natural home environment. (**C**) *Top*: Representative LFP spectral power recorded from the HPC. The characteristic spectral changes associated with awake and sleep behavioural states are visually evident with increased Delta frequency (1–4 Hz) activity in sleep and increased Beta frequency (13–20 Hz) activity in wakefulness. Simultaneous scalp PSG and LFP recordings were used to develop an automated LFP based behavioural state classifier: AWAKE, REM sleep and non-REM sleep (NREM 1, NREM 2 and NREM 3). *Bottom*: Hypnogram generated from continuous LFP recordings using a Naïve Bayesian Classifier. (**D)** Verbal memory tasks and mood assessments were performed remotely in participants’ home environment. ANT, anterior nucleus of thalamus; HPC-AMG, bilateral amygdala-hippocampus; IES, interictal epileptiform spikes; INSS, investigational implantable neural sensing and stimulation device; LFP, local field potentials; mTLE, mesial temporal lobe epilepsy; NREM, non-rapid eye movement sleep; REM, rapid eye movement sleep; Sz, seizure discharges. This figure was created by Mayo Clinic Media Illustrator and is permitted for use in this publication.

Continuous brain electrophysiology and dense behavioural data were collected from ambulatory participants using BrainRISE. The electrophysiology data,^35,39^ participant seizure-reports, anti-seizure medication, ambulatory verbal memory (word free-recall) testing^49^ and immediate mood scaler (IMS)^50,51^ data were collected using a mobile device running EPA, a custom software application enabling bidirectional communication between implanted devices, mobile devices and local and cloud computing resources. The EPA orchestrates communication between multiple wireless capable devices (sensing-stimulation implant, mobile devices, Apple Watch) and features custom automated Python narrow artificial intelligence algorithms for continuous analysis of long-term LFP data, control of electrical stimulation, impedance testing and LFP analysis. The EPA running on a mobile device provides an interface for performing verbal memory tasks, and collecting patient interactions^39,52^ (Fig. 2 and supplementary data). A key aspect of this platform is the application to monitoring ambulatory participants in their natural home environment, thereby capturing more realistic and ecologically relevant data.

#### Implantable neural sensing and stimulation device (INSS; supplementary data)

In this study, the investigational Medtronic Summit RC + S^TM^, a third-generation INSS, was integrated with the BrainRISE platform. The INSS provides bidirectional wireless communication, programmable 16-channel electrical stimulation, and continuous 4-channel (selected bipolar pairs) wireless LFP streaming.^39,53,54^ Bipolar pairs of contacts in ANT and AMG-HPC were used for sensing and streaming LFP (sampled at a frequency of 250–1000 Hz) and accelerometry data to the local mobile device running the custom EPA application. The electrode contacts used to create lead-specific bipolar recordings were selected by visual review of LFP data during seizures, resting wakefulness and sleep-wake transitions.

The INSS, electrical stimulation and embedded analytics^53^ do not require continuous connectivity with the BrainRISE system for therapy to remain active. The electrical stimulation therapy remains active as long as the implanted INSS device battery is adequately charged. During combined DBS and continuous 4-channel LFP streaming (250 Hz sampling) the INSS device requires daily charging. At 30% battery status, the LFP streaming is automatically disabled by the EPA application to maintain DBS therapy. In this scenario with LFP streaming disabled, DBS therapy remains active for ∼3 weeks. The system also supports duty cycle LFP streaming paradigms, e.g. 10 min every 60 min, that can be used to preserve battery charge.

#### Surgical implantation

Participants were anesthetized, fitted with a Leksell (Elekta) frame and underwent stereotactic MR imaging. The anterior commissure/posterior commissure was located using the COMPASS (COMPASS International) software, and the targets determined for deep brain stimulation (DBS) were applied using a Schaltenbrand and Wahren atlas overlay along with anatomical guidance, and direct visualization of mammillothalamic tract to identify the anterior nucleus of the thalamus (ANT).

Medtronic 3387 electrodes, as employed in the SANTE Trial, were inserted into the ANT, while Medtronic 3391 electrodes were placed along the medial temporal lobe's long axis. Bilateral hippocampal trajectory implants were used, designed to cover 2.5 cm of the HPC and 1 cm of the amygdala. Intraoperative fluoroscopy was utilized to ensure precise positioning. Once the leads were positioned, the patient was immediately moved (still under anesthesia) for a post-placement CT scan to confirm electrode localization. Upon verifying electrode locations, the patient was returned to the operating room, where the leads were tunneled to the INSS.

A single INSS was implanted in the left subclavicular area, and 60-cm pain lead (37087-60; Medtronic) extensions were utilized to achieve a 4-lead configuration with bilateral HPC and ANT. Leads from the right hemisphere pass across the skull and descend the neck on the left side, parallel to the left side lead implants.

Following implantation, the patient was transferred to the intensive care unit for recovery and was discharged on the first or second day post-surgery. During both the operation and intensive care unit stay, the device's sensing functions were examined, and data were collected.

#### MRI and diffusion tensor imaging

All participants had a seizure protocol 3 Tesla MRI and diffusion tensor imaging (DTI). Co-registration with post-op CT imaging was used to localize the electrodes within the ANT and AMG-HPC. Using frame stereotaxis bilateral ANT (Medtronic 3387 leads) and HPC & AMG (Medtronic 3391 leads) were implanted in 4 participants. Participant M2 was implanted with a 3387 rather than a 3391 in her residual left HPC tail. The residual left HPC tail was from a prior left anterior temporal lobectomy. The 3387 lead was used for its smaller contact spacing given the small posterior HPC tail remnant.

#### Lead and electrode contact localization

Five participants (M1, 2, 3, 4, 5) had four leads stereotactically implanted into bilateral ANT and bilateral AMG-HPC. Participant M5 does not have a right AMG electrode contact because the 3391-lead tail could not be fully seated into the lead extension connector at the time of surgery, leaving only 3 of the 4 contacts available for recording. For all participants 4 implanted leads (16 total electrode contacts) were localized with post-operative CT scan co-registered to the pre-op MRI and DTI for anatomic localization using previously described pipelines.^55,56^ The CT scan and electrode contact positions were co-registered to a T_1_ weighted anatomical MRI scan using SPM12 (https://fil.ion.ucl.ac.uk/spm/)^57^ Freesurfer (http://surfer.nmr.mgh.harvard.edu/) was used to segment the T_1_ weighted MRI and the electrodes labelled according to the Destrieux atlas.^58,59^ The final electrode contact localization for LFP and impedance analysis was performed with Lead DBS (see Supplementary material).^56^

#### Electrical stimulation of ANT

The ANT electrode pairs used for long-term ANT-DBS were selected by demonstrating the activation of Papez circuit, as evidenced by presence of a ∼40 ms latency HPC evoked response^60^ (see Supplementary material). Bipolar pairs were selected for chronic ANT-DBS to reduce stimulation artifacts and optimize HPC sensing for LFP seizure and IES detection (see supplementary data). Bipolar ANT electrode contact pairs were used for ANT-DBS with a continuous LF-DBS (2/7 Hz; 200 us pulse width; 2–6 mA) or duty cycle (1 min on/5 min off) HF-DBS (145 Hz; 100–200 us pulse width; 2–5 mA). Participants M1–M5 had periods of both LF- and HF-DBS, but M2 did not have adequate streaming data rates during HF-DBS and was omitted from the General Linear Mixed Model (GLMM) analysis (supplementary materials).

#### Long-term sensing LFPs

Four bipolar AMG-HPC electrode pairs or bilateral HPC and ANT were selected for sensing based on ability to record seizures and IES. The four bipolar pairs can be selected from any of the 16 electrode contacts. The LFP sampling rate can be set at 250, 500 or 1000 Hz. Most of the LFP data was collected at 250 Hz because of an increase in wireless data drops with higher 500 and 1000 Hz sampling rates. (See supplementary materials).

### Quantification and statistical analysis

#### LFP analysis

The LFP analysis was performed using the EPA and cloud platform with computational infrastructure and visualization. Validated automated machine learning algorithms were utilized to analyze prolonged LFP recordings for detecting seizures, IES^35^ and classifying Awake, REM and NREM^40,41,61^ brain states. This algorithmic pipeline is designed to detect seizures, IES, brain impedance,^62^ and to categorize sleep-wake stages in sequential 30-s data segments. Details on these algorithms and their efficiency were published previously^35,40^ (refer to supplementary materials). The sleep-wake classifications and seizure detections are aligned with patient reports for additional offline evaluation. The automated LFP analysis tools and data aimed at identifying IES, seizures and conducting sleep-wake classification are available at (https://github.com/bnelair/best_toolbox).

#### Seizure and IES detection

Seizures and IES serve as electrographic biomarkers of pathological, epileptogenic brain tissue, and are easily identifiable through human visual analysis of LFP recordings. We employed a previously validated algorithm^35,63^ to detect IES transients in LFP data. This adaptive IES algorithm facilitates the identification of IES within long-term LFP recordings, which often exhibit varying background activity typical in extended recordings over weeks to months. In this study, gold-standard training data was utilized to establish a hypersensitive threshold applicable to all participants (refer to supplementary materials).

The LFP associated with epileptic seizures show distinctive temporal and spectral changes covering a broad frequency spectrum. We have previously developed a precise seizure detection system using a convolutional neural network combined with long-short-term memory (CNN-LSTM), which takes the Short Time Fourier Transform of the LFP as its input.^35^ This CNN-LSTM model generates a seizure likelihood for every 10-s data segment. Seizures confirmed through a gold-standard visual review serve for training, validation and pseudo-prospective testing. The seizure detection effectiveness using the CNN-LSTM model was previously reported for participants M1-4 and is now extended to all five participants by applying an ultra-sensitive threshold to ensure comprehensive seizure detection for analysis. We manually examined all potential seizure events flagged by the highly sensitive automated CNN-LSTM detector. The daily temporal profile of seizures was analyzed by creating a circular histogram plotting the onset times of validated seizures for every participant.

#### Sleep-wake classification from continuous LFP recordings

Sleep-wake classification in ambulatory participants living in their natural home environment was performed using an individualized behavioural state classifier. As described previously in humans and canines, simultaneous LFP and gold-standard sleep annotations (expert review by EKS) from polysomnography (PSG) were used to train, test and validate the fully automated sleep-wake classifier.^40,41^ A validated Naïve Bayes classifier using features extracted from the LFP was used for long-term tracking of sleep-wake behavioural state classification.

#### Statistical analysis

**Figure 3:**
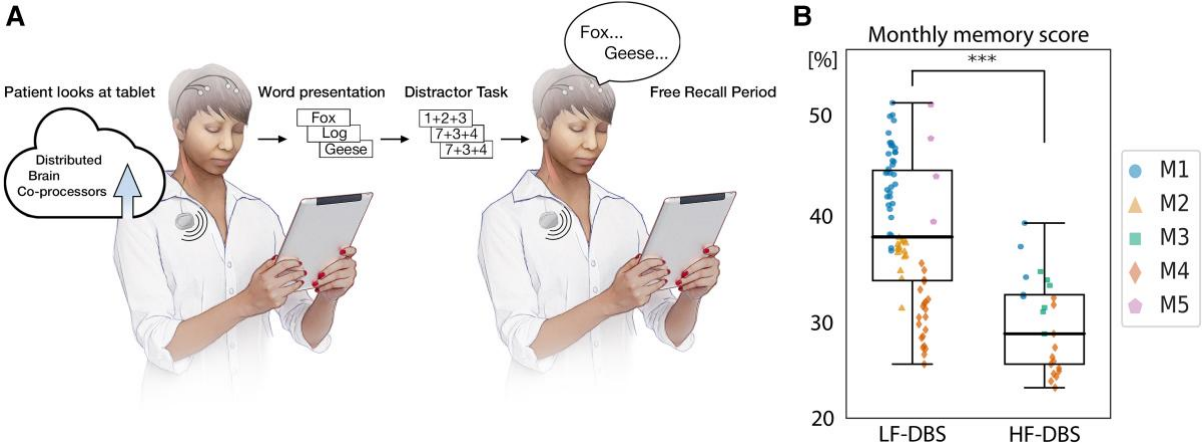
**Effect of ANT-DBS on verbal memory performance**. The impact of LF-DBS and HF-DBS ANT-DBS on verbal memory was investigated using a simple word encoding-recall task performed remotely by participants in their home environment. (**A**) Participants used the EPA application running on their mobile device to perform a validated word-recall task. The task was performed with the participant at different times of the day according to their individual preference. The task consisted of an average of 15 trials performed using lists of 12 words. A proper noun is randomly selected from a large database and presented on the screen for 10 s. After the presentation of the 12 nouns, the participant performs a simple math distractor. Subsequently, there is a period of free-recall where the participant recalls as many words, in any order, as possible from the prior encoding list. (**B)** Verbal memory scores for free-recall were better during periods where the participants were receiving LF-DBS compared with HF-DBS. Note, the ANT-DBS is off while the task is being performed. Data points represent monthly verbal memory score (percentage of words recalled) for individual participants: M1 (blue circle), M2 (yellow triangle), M3 (green square), M4 (red diamond), M5 (purple pentagon). Statistics: Two-sample Wilcoxon Rank Sum test (***P < 0.001).

We used a two-sample Wilcoxon Rank Sum test to compare the effect of LF-DBS and HF-DBS on memory and sleep. For analysis of the effect of DBS on memory (Fig. 3), we compare the words recalled in the two DBS conditions, (LF-DBS and HF-DBS). For analysis of the effect of DBS on sleep, we compare the wake after sleep onset (WASO) and duration of rapid eye movement (REM) and non-REM in the three DBS stimulation conditions (No-stim, LF-DBS and HF-DBS).

For the analysis of seizures, IES and DBS, we use the R statistics package^64^ to create generalized linear mixed models (GLMMs) with participants included as the grouping factor in the random effects of the GLMM. This is done to account for the correlation between repeated measures within individuals with observed high variability in the response variables between participants.

**Figure 4:**
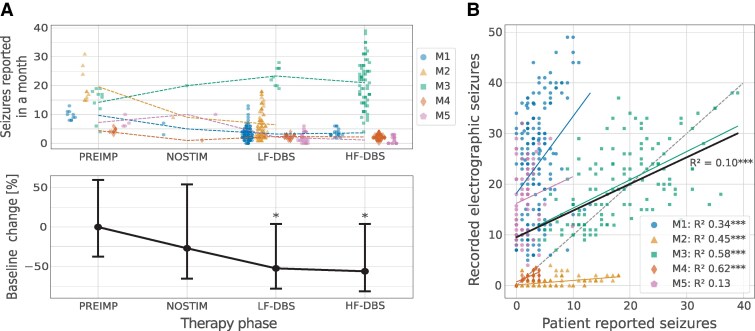
**ANT-DBS and seizures**. Participants reported their seizures using the EPA running on a mobile device during baseline prior to surgery (PREIMP: 3 months prior to implant), after device implant before starting ANT-DBS (NOSTIM: 4 weeks), and during LF-DBS and HF-DBS. (**A)***Top*: Scatter plot of individual monthly reported seizures and the mean over all months (dashed lines). *Bottom*: Both LF-DBS and HF-DBS reduced reported seizures compared with baseline by over 50% at a group level and in four of five individuals. The participant M4 with an increase in reported seizures after implant was receiving high-dose diazepam therapy that had to be discontinued because of mood decline and fatigue on week 5 after implant, which may have precipitated an increase in seizures. (**B**) Participant reported seizures versus detected electrographic seizures captured with continuous hippocampal LFP streaming. Notably, some participants reported seizures (e.g. M3) that were not associated with an electrographic LFP correlate and some (M1 and M5) had more electrographic LFP seizures than they reported. Statistical Analysis: A GLMM with participants included as the grouping factor in the random effects. The GLMM group estimated fit with confidence intervals in solid black. (*P < 0.05 and ***P < 0.001).

In the model for self-reported seizures, the categorical predictor included study periods (Baseline, NOSTIM, LF-DBS, HF-DBS), and the response variable was the number of self-reported seizures over a 4-week period. The model included all four study periods as both fixed and random effects. The R formula used in this GLMM for this model (Fig. 4) is:


SelfReportedSeizures∼StudyPeriod+(StudyPeriod|Patient),

where StudyPeriod is a categorical variable representing the study periods (Baseline, NOSTIM, LF-DBS, HF-DBS), and the random effects are again specified in parentheses. Patient is the grouping variable for individual patients (M1−M5).

In the model for electrographic seizures, the categorical predictor is the stimulation study period (LF-DBS and HF-DBS). We consider the fixed and random effects of low-frequency DBS and high-frequency DBS (LF-DBS versus HF-DBS) on seizures. The R formula used in the GLMM for this model is:


iEEGSeizureCount∼StudyPeriod+(StudyPeriod|Patient),

where StudyPeriod is a categorical variable representing LF-DBS and HF-DBS, and the random effects are specified in parentheses. Patient is the grouping variable for individual patients (M1–5).

#### Verbal memory assessment in ambulatory subjects in a natural home environment

Verbal memory was tracked in ambulatory participants using an established verbal memory paradigm.^49,65,66^ The task was programmed on the EPA application (Figs 2D and 3A) and remotely performed with the participant at different times of the day during the week, at their preference. The ANT-DBS was turned off during the task. Investigators remotely activated the task on a mobile device, recorded and documented the verbal responses. In this task, participants were presented with a list of 12 proper nouns with the goal of later recalling them. Participants were instructed to commit these individual words to memory as they appeared one by one on a mobile device screen. Each word was displayed for a duration of 1600 ms, followed by a randomly varying blank interval of 750–1000 ms between words.

Immediately after the presentation of the final word in each list (during the encoding phase), participants engaged in a 20 s distractor task consisting of solving arithmetic problems. Following the completion of the distractor task, participants were tasked with verbally recalling as many of the twelve words as they could from the previously presented list, in any order, within a 30-s time frame (recall phase). Each session encompassed 25 sets of this encoding-distractor-recall procedure. The stimulation was reactivated upon successful completion of the task. Tasks performed after recent seizures were discarded.

### Immediate mood assessments

Mood assessments were integrated into the mobile EPA, which was programmed to randomly query participants for their responses. Participants were prompted on a randomly selected day and time (10 a.m.–6 p.m.; one-to-four times a week) to complete the Immediate Mood Scaler 12 (IMS).^50^ The IMS is an ecological momentary assessment of 12 questions (7-point Likert scale) evaluating anxiety and depression symptoms in the moment. (See supplementary materials).

## Results

All seven participants enrolled had mTLE and reported comorbid depression, anxiety and sleep disturbances (insomnia, hypersomnolence). Participants underwent comprehensive Phase-I non-invasive evaluations for their drug-resistant epilepsy, including MRI, DTI, functional imaging and multi-day video scalp EEG. Three participants had invasive intracranial stereo-EEG. Six participants met the inclusion criteria with at least three reported disabling seizures per month at baseline using the mobile application to create an electronic diary of patient-reported seizures. One participant declined further participation after the baseline data collection despite meeting all inclusion criteria. Five participants were implanted with an investigational INSS device (Fig. 2 and Supplementary material).

### Patient-reported outcomes and system related adverse events

The custom EPA application running on a mobile device was used by participants to report their seizures. When compared with baseline reported seizure counts, the patient-reported seizures were reduced with both LF-DBS and HF-DBS stimulation (Fig. 4A; *P* < 0.05). There was no difference in the reduction of the patient-reported seizures when directly comparing LF-DBS and HF-DBS (−52% for LF-DBS and −56% for HF-DBS).

Participants reported an average of 162 *±* 158 seizures over the course of the study, with variable discrepancies between patient-reported seizures and reports with LFP correlates. We directly compared reported seizures with detected electrographic seizures^35^ (Fig. 4B). There was a correlation between reported seizures and detected electrographic seizures on a group level, with substantial variability across participants (overall *R*^2^ = 0.1, *P* < 0.001). However, participants variably reported seizures without LFP correlates and under reported detected electrographic seizures (Fig. 4B). Participant M4 had the highest correlation between electrographic LFP-detected seizures and patient-reported seizures (*R*^2^ = 0.62, *P* < 0.001). This is in contrast with the absence of correlation in participant M5 (*R*^2^ = 0.13, *P* > 0.05). It is noteworthy that although participant M4 was amnesic for her focal impaired awareness seizures (FIAS), her spouse was often able to accurately log these episodes. This was made possible due to close observation during work-from-home arrangements in the COVID-19 lockdown.

In the five mTLE participants implanted with the INSS, comprehensive mood, quality of life, seizure severity, and NP testing were collected during in-person clinic visits at pre-implant baseline, 3–6 months post-implant, and at 9–15 months post-implant. There were no significant differences across participants and timepoints for the standard measures of mood, quality of life, seizure severity, or NP testing (supplementary materials).

All participants had NP testing at baseline and during LF-DBS and HF-DBS. Participant M3 had a decrease in Verbal Paired Associates (VPA) immediate and delayed recall compared with baseline. For M5, both Rey Auditory Verbal Learning Test and VPA immediate recall were reduced during high-frequency ANT-DBS. These changes did not reach significance.

Four device-related adverse events were reported over the 15-month protocol. Participant M2 inadvertently disabled therapy using the EPA application. A subsequent EPA reversion corrected this potential for operator mistake, and the cloud dashboard more clearly displayed the therapy status to the clinical team. Two participants (M1 and M4) experienced behavioural changes with anxiety, dysphoria and sleep disturbance during trials of continuous HF-DBS (see supplementary data). In both participants, the reported anxiety, dysphoria and sleep disturbance resolved with changing from continuous to duty cycle (1 min on and 5 min off; SANTE parameters^17^) HF-DBS. In participant M5, the right amygdala-hippocampal lead could not be fully seated into the lead extension connector at the time of surgery, leaving only three of the four contacts recording. There were no deaths, infections, bleeding, strokes, status epilepticus, or other device-related complications.

Subject M4 was hospitalized for whole-body paralysis and unresponsiveness lasting hours that did not have any LFP correlate and was determined to be consistent with a non-epileptic spell. Subject M5 had three overnight hospitalizations with recurrent seizures like his baseline.

### Seizures and IES and their circadian patterns

**Figure 5:**
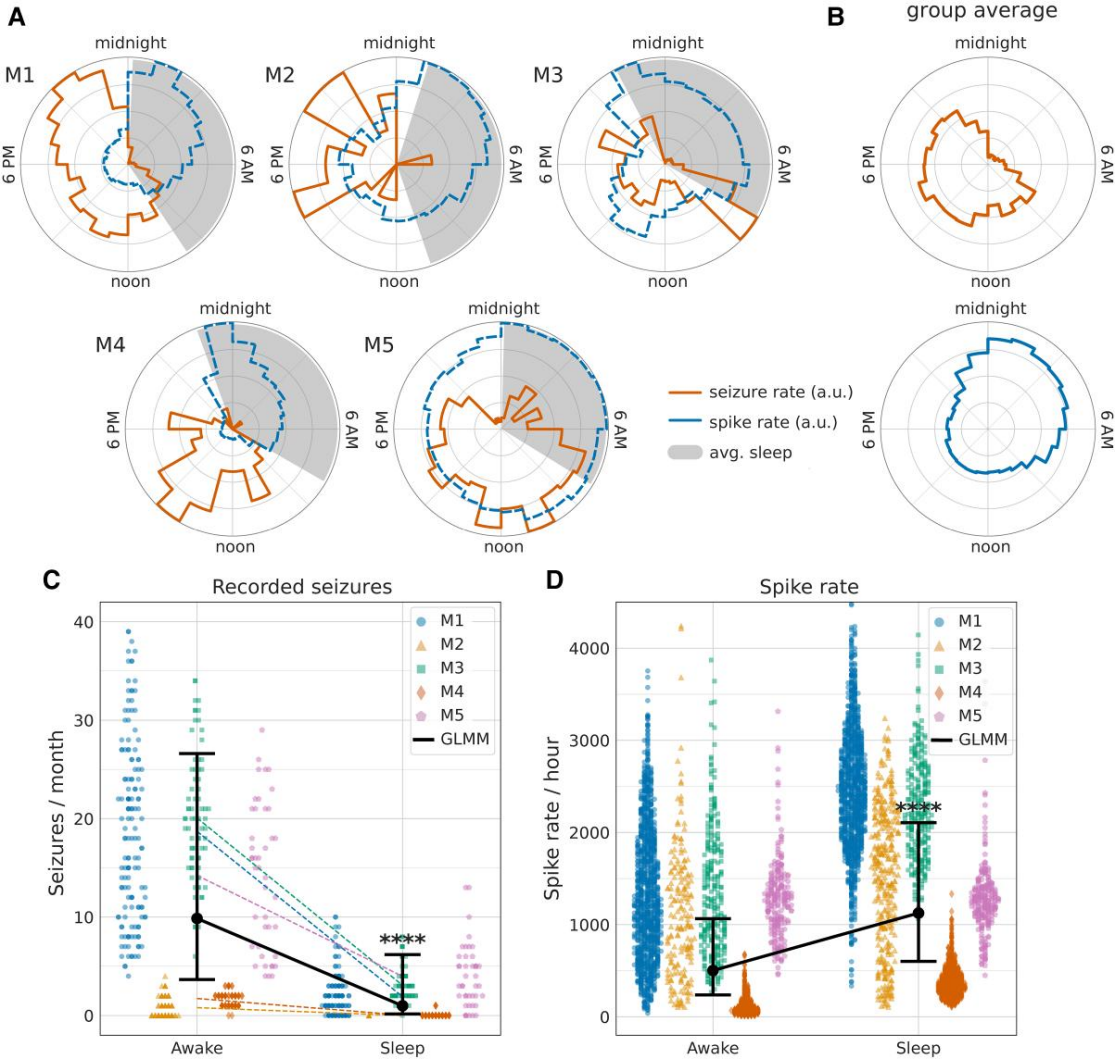
**Circadian patterns of seizures and IES**. Epileptogenic human HPC generates spontaneous Sz and IES that are modulated by sleep-wake behavioural states. Habitual Sz primarily occurred during wakefulness with a complex, participant specific, distribution of increased occurrence. Seizures rarely occurred during sleep compared with wakefulness. Conversely, IES were increased during sleep, particularly in non-rapid eye movement (NREM). (**A**) Circular 24-h histograms of Sz occurrence for each participant show detected electrographic Sz (red-solid), IES rates (blue-dashed) and sleep-wake classification (grey-filled) using validated algorithms applied to continuous LFP. For all participants the Sz primarily occur during wakefulness with patient specific distributions approximating unimodal (M4 and M5) or bimodal (M1,2,3) daytime distributions. (**B**) Aggregated Sz and IES across all participants show the differential behavioural state modulation of epileptiform activity. (**C**) Recorded seizures/month for M1 (blue circle), M2 (yellow triangle), M3 (green square), M4 (red diamond), M5 (purple pentagon) for each participant. The seizures are more common in daytime 9.86 (95% CI: 3.65–26.62) seizures/month during wakefulness versus sleep 0.99 (95% CI: 0.16–6.18) seizures/month. (**D)** IES rates (spikes/hour) are higher in sleep 1125.25 (95% CI: 601.50–2105.62) spikes/hour compared with wake 502.11 (95% CI: 236.87–1064.35) spikes/hour. Statistical analysis for (**C**) and (**D**): A GLMM with patients included as the grouping factor in the random effects. The GLMM group estimated fit with confidence intervals in solid black. (****P < 0.0001).

All participants had independent left and right temporal lobe IES and seizures. Characterization of long-term LFP recordings showed that electrographic seizures and IES occurred with greater frequency in the left HPC in each of the five participants. All participants had day-night patterns of epileptiform activity, with IES maximal at night during sleep and seizures maximal during the day during wakefulness (Fig. 5). The seizures captured from continuous LFP recordings demonstrated a strong diurnal pattern with most seizures occurring during the daytime [1366 seizures during wakefulness versus 186 seizures during sleep (*P* < 0.0001), Supplementary Table 10]. The timing of the daytime seizures was patient-specific, with M4 showing a tendency for seizures at 2:30 p.m. ± 1 h, two participants (M1, M3) with seizures preferentially occurring in the morning and evening, M2 with late afternoon and evening seizures, and M5 with a more uniform distribution of seizures over the day.

The pattern of IES over a 24-h period is distinctly different from seizures. For all participants, the IES rates are increased during sleep compared with wake, and seizures are more common during the day. (Figure 5B and C and see Supplementary material).

### Impact of ANT-DBS on IES and detected seizures

**Figure 6:**
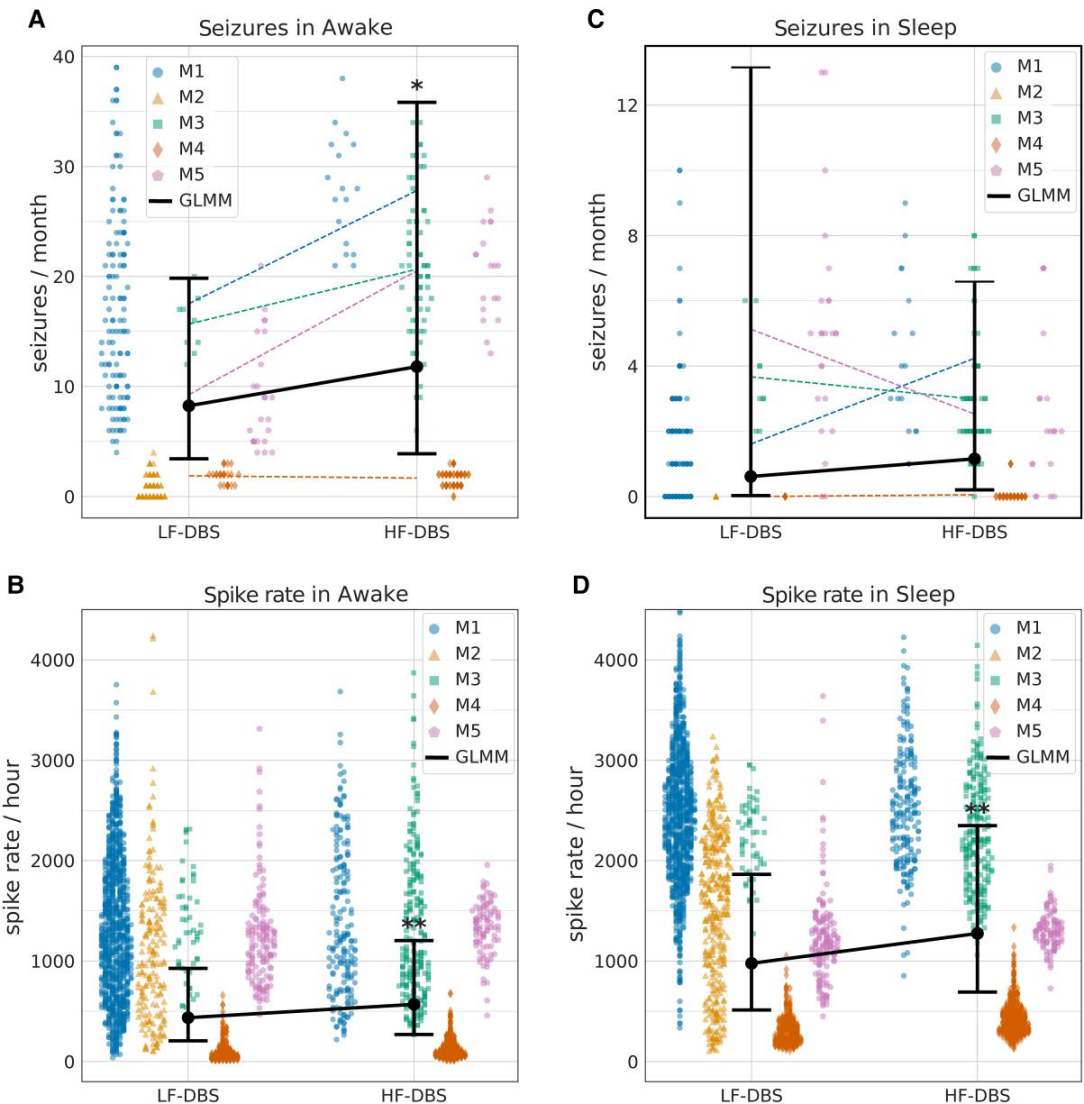
**Electrographic seizures and IES recorded from HPC during ANT-DBS**. Comparison of LF-DBS and HF-DBS stimulation paradigms. (**A**) The electrographic seizures during wakefulness recorded from bilateral HPC were reduced during continuous LF-DBS 8.24 (95% CI: 3.43–19.83) seizures/month compared with duty cycle HF-DBS 11.80 (95% CI: 3.88–35.83) seizures/month. (**B**) The IES rates during wakefulness were decreased during LF-DBS 435.75 (95% CI: 205.03–952.11) spikes/hour compared with HF-DBS 568.46 (95% CI: 268.71–1202.60) spikes/hour. (**C**) Electrographic seizures during sleep recorded from bilateral HPC are infrequent and occur with similar rate during LF-DBS 0.61 (95% CI: 0.03–13.15) seizures/month and HF-DBS 1.15 (95% CI: 0.20–6.58) seizures/month. (**D**) The IES rates during sleep were decreased during LF-DBS 976.54 (95% CI: 512.23–1861.73) spikes/hour compared with HF-DBS 1273.96 (95% CI: 690.77–2349.50) spikes/hour. In each panel the data points represent seizures/month (**A**), (**C**) and spikes/hour (**B**), (**D**) for individual participant: M1 (blue circle), M2 (yellow triangle), M3 (green square), M4 (red diamond), M5 (purple pentagon). Statistical Analysis: A GLMM with participants included as the grouping factor in the random effects. The GLMM group estimated fit with confidence intervals in solid black. (*P < 0.05, **P < 0.01).

Unlike patient-reported seizures, where there was no difference between LF-DBS and HF-DBS, there was a difference when considering electrographic seizure and IES. The catalog of detected seizures using streaming LFP showed that LF-DBS reduced seizures more than HF-DBS (*P* < 0.05) in wakefulness (Fig. 6A). Furthermore, LF-DBS reduced IES compared with HF-DBS during both sleep and wakefulness (*P* < 0.01) (Fig. 6B and D). Seizures detected from streaming LFP were similar for HF-DBS and LF-DBS during sleep, but the number of seizures during sleep was small (186) compared with wakefulness (1366).

### Impact of ANT-DBS on sleep

The LFP recordings from HPC were used as input to a validated, individualized, automated Naive Bayes sleep-wake state classifier^40^ (see Materials and methods and supplementary data). As participants slept ad libitum on their own preferred sleep-wake schedules, characteristics of sleep varied widely with a range of individual values for sleep onset, offset and duration. The number of NREM-REM cycles demonstrated substantial intra-individual and inter-individual variability. On average, across all participants, the total sleep duration was 8 h. 33 min, with 4.04 NREM-REM cycles and 19.9 min NREM epoch duration.

**Figure 7:**
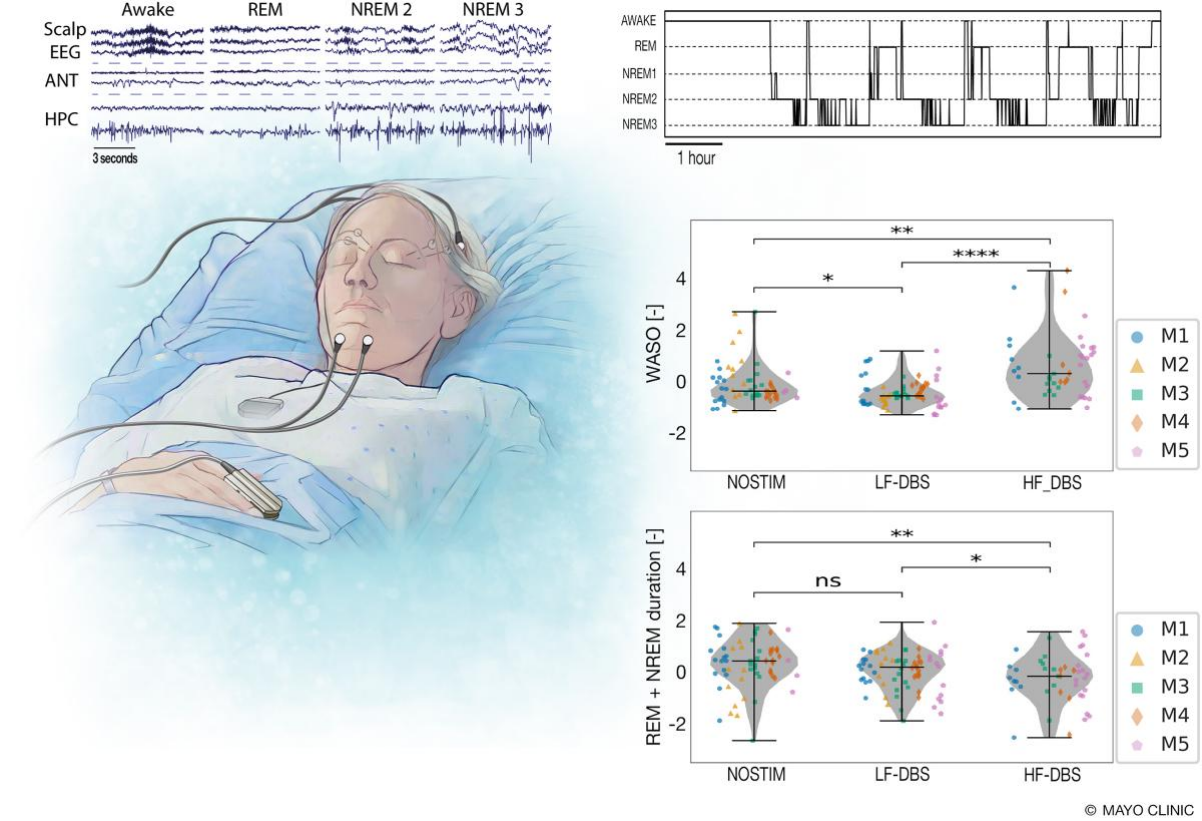
**Effect of ANT-DBS on sleep**. The impact of ANT-DBS neuromodulation on sleep was investigated using long-term behavioural state classifications (sleep-wake) from a validated Naïve Bayesian Classifier applied to LFP recorded from the HPC of ambulatory participants in their home environments. Top left: Simultaneous PSG (Scalp EEG, from the *top*: channels Fz-TP11, Cz-TP11 and Oz-TP11) and streaming intracranial LFP recordings from right (*top*) and left (*bottom*) ANT and right (*top*) and left (*bottom*) HPC are used for training and testing of the automated sleep-wake classifier. Top right: An example of a gold-standard hypnogram during simultaneous PSG and intracranial LFP recording. Similar hypnograms were obtained for multiple months using only intracranial LFP in participants in their home environments. The automated sleep-wake classifier labelled behavioural states as Wakefulness (AWAKE), REM sleep and non-REM sleep (NREM: NREM 1, NREM 2 and NREM 3). Bottom right: The time spent WASO was reduced by LF-DBS compared with baseline without ANT-DBS. The HF-DBS increased WASO compared with both baseline and LF-DBS. The overall time spent in NREM and REM was reduced during HF-DBS compared with the no stimulation baseline and LF-DBS. The total NREM-REM time in sleep with LF-DBS was indistinguishable from the no stimulation baseline and longer than HF-DBS. Data points represent contribution of individual participants: M1 (blue circle), M2 (yellow triangle), M3 (green square), M4 (red diamond), M5 (purple pentagon) in given condition (NOSTIM—without ANT-DBS stimulation, LF-DBS, HF-DBS). Statistics: Two-sample Wilcoxon Rank Sum test (*P < 0.05, **P < 0.01, and ****P < 0.0001). This figure was created by Mayo Clinic Media Illustrator and is permitted for use in this publication.

ANT-DBS neuromodulation with LF-DBS and HF-DBS did not impact total sleep duration (sleep offset—onset), but the time spent WASO was reduced by LF-DBS compared with baseline without stimulation (*P* < 0.05). In contrast, HF-DBS increased WASO compared with baseline and LF-DBS (*P* < 0.001). The overall time spent in NREM and REM was decreased during HF-DBS compared with baseline without stimulation and LF-DBS. The total NREM-REM time in sleep with LF-DBS neuromodulation was indistinguishable from baseline without ANT-DBS and longer than HF-DBS (*P* < 0.05). (Figure 7 and supplementary materials).

### Impact of ANT-DBS on memory (ambulatory free-recall task)

Participants used the EPA application on a mobile device to remotely perform a validated word free-recall task^49,65,67,68^ in their natural home environment (Fig. 3A). All participants performed the ambulatory free-recall memory task after implant of the ANT-DBS system. The ambulatory free-recall verbal memory task was not functional on the EPA system until after the time of implant and pre-implant data were not captured. The task was performed with the subject at different times at the participant’s preference (hour, day of week) over multiple months during the two ANT-DBS stimulation paradigms. However, as noted previously, all participants had standard verbal memory testing that did not show a change comparing baseline prior to implant, LF-DBS and HF-DBS. The free-recall verbal memory scores were higher during LF-DBS compared with HF-DBS (*P* < 0.001; Fig. 3B) (See supplementary materials).

### Impact of ANT-DBS on mood

All participants completed assessments of mood (Beck Depression Index) and suicidality (Columbia Suicide) during baseline, LF-DBS and HF-DBS. In addition, during LF-DBS and HF-DBS, participants were prompted at random times of the day to complete the Immediate Mood Scaler 12 (IMS)^50^ deployed on their mobile device (EPA application) to densely track mood and anxiety symptoms instantaneously in their naturalistic environment (see supplementary materials). Symptoms of depression and anxiety were present in all five participants, but there were no differences detected between HF-DBS and LF-DBS for mood and anxiety symptoms (see supplementary materials).

In the initial subject, M1, a trial of continuous HF-DBS was associated with side effects that improved after transition to duty-cycle high-frequency DBS (HF-DBS). Similarly, in subject M4 during a hospital stay in the EMU, we tested continuous HF-DBS with side effects. We recently described the side effects of continuous HF-DBS in more detail.^69^ We subsequently only used duty-cycle for HF-DBS, similar to what was used in the original SANTE trial.^17^

## Discussion

Epilepsy is a complex disorder where the impact of treatment on quality of life depends on seizures outcomes as well as comorbidities. Here we investigated five people with drug-resistant mTLE to focus attention on limbic network circuits generating disabling, drug-resistant seizures and MMS comorbidities. We created a neurotechnology platform (BrainRISE) integrating implantable and mobile devices within a cloud infrastructure to investigate the impact of ANT-DBS therapy on seizures, IES and MMS. Synchronized data streams^69^ from multiple devices (brain sensing-stimulation implanted device, mobile computing and wearable devices) were streamed to a cloud-based data storage, viewing and computing environment. Validated algorithms were applied to streaming hippocampal LFP recordings to create accurate catalogs of seizures, IES and sleep-wake behavioural states. Subjects also reported seizures and performed a word free-recall memory task and answered mood questionnaires using the custom mobile application. The results demonstrate that the participants are often poor reporters of their seizures. This has been demonstrated previously by multiple groups using implanted devices^32,34^ and in the EMU.^32,70^ Patients may attribute various symptoms to seizures despite a non-epileptic etiology and without closed-loop responsiveness testing, it remains unclear what patients are experiencing. We have recently demonstrated automated responsiveness testing in epilepsy that we plan to use in future studies to correlate patient behaviour and electrographic seizures.^71^ The current study demonstrates the feasibility and potential clinical utility of continuous, synchronized tracking of brain electrophysiology and behaviour in PWE living in their natural home environments (Fig. 2).

We characterized the circadian patterns of epileptiform activity in mTLE and showed that seizures occur primarily during wakefulness and IES are markedly increased during sleep, as has been previously reported in studies with more limited data.^72-74^ The mechanism underlying the discordance of behavioural state-dependent IES and seizures, two fundamental measures of pathological brain excitability, remains unclear. We speculate that behavioural state-dependent modulation of limbic network circuits differentially effects activation, spread and network propagation of pathological hippocampal electrographic activity to reduce seizures during NREM and REM sleep compared with wakefulness.

We quantified the effects of ANT-DBS on mTLE epileptiform activity and surprisingly show that LF-DBS is as effective as the widely utilized, and FDA and Ce-mark approved HF-DBS for reducing patient-reported seizures in this small group of patients. But possibly more important, LF-DBS showed greater reductions of electrographic seizures and IES (Figs 4–6), objective biomarkers of pathologic network excitability,^75-77^ in both wakefulness and sleep. Furthermore, LF-DBS was associated with improved objective measures of sleep (Fig. 7) and verbal memory (Fig. 3B) compared with HF-DBS.

HF-DBS is proven to reduce seizures,^17^ but there may be more optimal stimulation paradigms depending on the circuits and etiologies underlying the epilepsy, seizures and MMS comorbidities. Here we show that in a small number of well-characterized mTLE participants having hippocampal onset seizures, LF-DBS was more effective in reducing epileptiform activity and was better for sleep and memory than HF-DBS. We can speculate that the reduced NREM-REM duration is likely related to arousals. This is supported by the results showing that WASO is increased with HF-DBS (Fig. 7).

The clinical relevance of reducing IES and subclinical electrographic seizures in epilepsy has long been debated, but recent studies show the deleterious impact of IES on memory^78-81^ and sleep.^82^ Furthermore, the role of IES on progression and maintenance of epilepsy remains poorly understood, but IES in slow-wave sleep may play a role in disruption of long-term memory^65^ and consolidation of seizure engrams and epileptogensis.^83,84^ It is important to note that participants receiving ANT-DBS, both LF-DBS and HF-DBS, over multiple months did not show evidence for kindling with increasing IES and seizures.^84^ In the future, the BrainRISE platform can be used for optimizing ANT-DBS paradigms, but results will first require replication and validation in a larger cohort of patients.

While our current study focused on establishing the integrated platform in a small number of subjects, we would like to highlight the potential for LFP biomarker-guided DBS, particularly in the context of multi-node ANT-HPC stimulation. Future investigations will explore closed-loop paradigms utilizing this platform to personalize and optimize DBS therapy based on individual patient's LFP and behavioural biomarkers.

There are several limitations in this study. (i) The trial design was focused on safety and feasibility and does not include blinding or randomization of a control group without ANT-DBS therapy activated. Given the proven, class-I evidence for ANT-DBS, we did not feel there was clinical equipoise to withhold ANT-DBS therapy from patients for an extended period. We did not control for duration of the therapy periods, but comparing equal reporting periods did not affect the results. (ii) The existence of cycles of seizure occurrence in epilepsy^72^ is now well established and the results obtained in any future trial will likely depend on the timing of therapy and data collection. We have largely addressed this limitation here with the dense long-term monitoring. (iii) Similar to other studies, we use patient reports of seizures to assess the outcome of ANT-DBS. There is strong evidence that many PWE are poor reporters of their seizures. This was also demonstrated in our data when comparing proven electrographic seizure discharges with patient reports. This remains a fundamental limitation in clinical epileptology, but here the gap is partially mitigated by the unique long-term continuous LFP sensing and automated seizure detection after device implant. (iv) Similar to the clinical trial leading to regulatory approval of high-frequency ANT-DBS, we did not see differences in standard NP and memory testing during in person clinic visits at baseline prior to implant and during both LF-DBS and HF-DBS. But with the ambulatory free-recall memory and IMS tasks, we were not able to obtain baseline assessments prior to implant. Unfortunately, the software system was not completed prior to enrollment of participants. Therefore, we could only compare differences in memory free-recall tasks and IMS scores after implantation between LF-DBS and HF-DBS. (v) The BrainRISE platform provides synchronized behaviour and neural activity on an unprecedented scale in ambulatory humans, but challenges remain given the participant burden associated with keeping multiple device batteries charged and wireless connectivity active. (vi) It remains unknown if, and to what degree, electrographic seizures detected from streaming LFP impair consciousness. This is a fundamental gap in the assessment and treatment of epilepsy that will be addressed in the future with assessments that are triggered on seizure detections in real time.^85-89^

## Supplementary Material

fcaf106_Supplementary_Data

## Data Availability

The Bioelectronics Neurophysiology and Engineering Lab is committed to the practice of sharing data and code to create reproducible research. The data will be shared on reasonable request to the corresponding authors. The code is available on GitHub through links from BNEL lab webpage: https://www.mayo.edu/research/labs/bioelectronics-neurophysiology-engineering/data-code-sharing.
